# Efficacy of cognitive behavioral therapy on aggressive behavior in children with attention deficit hyperactivity disorder and emotion dysregulation: study protocol of a randomized controlled trial

**DOI:** 10.1186/s13063-022-05996-5

**Published:** 2022-02-07

**Authors:** C. Vacher, L. Romo, M. Dereure, M. Soler, M. C. Picot, D. Purper-Ouakil

**Affiliations:** 1 Centre Hospitalo-Universitaire de Montpellier, Service Médecine Psychologique de l’Enfant et de l’Adolescent, Montpellier, Hérault France; 2 CLIPSYD EA-4430, UFR Sciences Psychologiques et Sciences de l’Education, Université de Nanterre, Nanterre, Hauts de Seine France; 3 INSERM U 1018, CESP, Psychiatrie du développement – Evaluer et traiter les troubles émotionnels et du neurodéveloppement (ETE-ND), Montpellier, France; 4 Service de Pathologies professionnelles et de l’environnement, Assistance Publique des Hôpitaux de Paris, Hôpital Universitaire Raymond Poincaré, Garches, France; 5 Unité de Recherche Clinique et Epidémiologie, Département de l’Information Médicale, Centre Hospitalo-Universitaire de Montpellier, Montpellier, Hérault France; 6 Centre d’Investigation Clinique, Hôpital Saint Eloi, Centre Hospitalo-Universitaire de Montpellier, Montpellier, Hérault France

**Keywords:** ADHD, Emotion dysregulation, Children, Cognitive behavioral therapy

## Abstract

**Background:**

Attention deficit hyperactivity disorder (ADHD) is frequently associated with emotional dysregulation (ED). ED is characterized by excessive and inappropriate emotional reactions compared to social norms, uncontrolled and rapid shifts in emotion, and attention focused on emotional stimuli. Few studies have evaluated non-pharmacological interventions to improve ED in children with ADHD. The current randomized controlled trial assesses the efficacy of a cognitive behavioral therapy (CBT) intervention compared with a theater-based intervention (TBI) in children with ADHD and ED.

**Methods:**

Sixty-eight 7- to 13-year-old children with ADHD and ED will be recruited and randomly assigned to the CBT or TBI group. CBT aims to reduce ED by teaching anger management strategies. TBI seeks to reduce ED by improving emotion understanding and expression through mimics and movement. In both groups, children participate in 15 1-h sessions, and parents participate in 8 sessions of a parent management program. The primary outcome measure is the change in the “Aggression” sub-score of the Child Behavior Checklist (CBCL). Secondary outcome measures include overall impairment (Children’s Global Assessment Scale, Strengths and Difficulties Questionnaire), personality profile (Hierarchical Personality Inventory for Children), executive function (Behavioral Rating Inventory of Executive Function), quality of life (Kidscreen-27), parental stress (Parenting Stress Index, 4th edition), parental depression (Beck Depression Inventory-II), and impact of child disorders on the quality of the family life (Parental Quality of Life and Developmental Disorder).

**Discussion:**

Children with ADHD and ED are at risk of functional impairment and poor outcomes and have specific therapeutic needs. This randomized controlled trial wants to assess non-pharmacological treatment options for this population.

**Trial registration:**

Clinicaltrials.gov. NCT03176108. Registered on June 5, 2017.

## Administrative information

Note: the numbers in curly brackets in this protocol refer to SPIRIT checklist item numbers. The order of the items has been modified to group similar items (see http://www.equator-network.org/reporting-guidelines/spirit-2013-statement-defining-standard-protocol-items-for-clinical-trials/).

**Table:** 

Title {1}	Efficacy of Cognitive-Behavioral Therapy (CBT) on aggressive behavior in children with ADHD and emotion dysregulation: study protocol of a randomized controlled trial
Trial registration {2a and 2b}.	Clinicaltrials.gov. Number: NCT03176108. Registered on June 5, 2017
Protocol version {3}	August 8, 2018; version n. 3
Funding {4}	The trial is funded by Montpellier University Hospital, France
Author details {5a}	Vacher, C^1,2,3^., Romo, L^2,5^.,Dereure, M^4^., Soler, M^4^., Picot, M.C^4,6^., & Purper-Ouakil, D^1,3^ 1 Service Médecine Psychologique de l’Enfant et de l’Adolescent, Centre Hospitalo-Universitaire de Montpellier, Montpellier, Hérault, France 2 CLIPSYD EA-4430, UFR Sciences Psychologiques et Sciences de l’Education, Université Paris Nanterre, Nanterre, Hauts de Seine, France 3 INSERM U 1018, CESP, Psychiatrie du développement – Evaluer et traiter les troubles émotionnels et du neurodéveloppement (ETE-ND), Montpellier, France 4 Unité de Recherche Clinique et Epidémiologie, Département de l’Information Médicale, Centre Hospitalo-Universitaire de Montpellier, Montpellier, Hérault, France 5 Service de Pathologies professionnelles et de l’environnement, Assistance Publique des Hôpitaux de Paris, Hôpital Universitaire Raymond Poincaré, Garches, France 6 Centre d’Investigation Clinique, Hôpital Saint Eloi, Centre Hospitalo-Universitaire de Montpellier, Montpellier, Hérault, France
Name and contact information for the trial sponsor {5b}	Vacher Cécile c-vacher@chu-montpellier.fr
Role of sponsor {5c}	The sponsor of this trial is Montpellier University Hospital and its team collects, analyzes and interprets the data; and writes the report in collaboration with Prof Romo. The decision to submit the report for publication comes from the principal research investigator.

## Introduction

### Background and rationale {6a}

Attention deficit hyperactivity disorder (ADHD) is a complex neurodevelopmental disorder with a prevalence estimated at 5.3% over the years [9, 68]. ADHD is characterized by high levels of inattention, hyperactivity, and impulsivity [6] that have a lasting impact on the child and family’s life.

Significant emotion dysregulation (ED) is observed in 24 to 50% children with ADHD [69, 76] and in 30 to 70% of adults with ADHD [69]. ED is the inability to modulate emotional responses in a given context [85, 88], and includes (a) excessive and inappropriate emotional reactions and experiences relative to social norms; (b) uncontrolled and rapid shifts in emotions; and (c) abnormal allocation of attention to emotional stimuli [76]. ED clinical expression is characterized by mood instability, irritability, aggression, tantrums [55], and low tolerance to frustration [22, 62, 95]. Definitions of ED tend to focus only on negative emotions, but children with ADHD and ED can also experience difficulties in regulating positive emotions [26, 77]. Physiological arousal (gritting teeth, upturned mouth, physical tension…) and cognitive biases (“Why is that person strangely looking at me?”; “He/she purposely annoys me”) contribute to the socially inappropriate behaviors in patients with ED [72]. As ED is present in many psychiatric disorders (e.g., anxiety disorders, mood disorders, neurodevelopmental disorders), it is not specific to ADHD [21, 43, 81].

Besides the core ADHD symptoms, children with ADHD and ED show higher levels of functional impairment [71, 90], aggressive behaviors, oppositional defiant disorder, mood and anxiety disorders [7, 89]. Children with ADHD and ED use inappropriate coping strategies that interfere with their academic performance and social functioning [90]. Therefore, ED contributes to their social and academic difficulties [21, 78, 83].

Aggressive behaviors are one of the most problematic expressions of high ED [7, 53]. Aggression is defined as verbal and/or physical acts with the aim of harming another person, directly or indirectly [36]. Proactive aggression is a deliberate goal-oriented behavior that is motivated by external reinforcements [20, 25]. Reactive aggression is a violent reaction to ambiguous situations where the behavior of others tends to be interpreted as hostile and threatening [25, 26]. Reactive aggression is frequent in ADHD and is associated with difficulties of self-regulation, including ED and executive function deficits [11, 20, 65, 96]. Children with ADHD and high levels of aggression also show more inadequate emotional coping strategies compared with those with low levels of aggression [63].

Medications, such as psychostimulants and risperidone, improve aggressive behaviors, emotional dysregulation, and rageful outbursts ([15, 18]; Fernández [28, 35, 46, 84, 86, 97]). It has been hypothesized that such drugs, by improving impulsivity, allow children with ADHD to better understand emotions and to plan appropriate strategies to cope with problematic situations [84].

Many studies have also focused on psychosocial interventions (parental management training, CBT…) of irritability, but not specifically in children with ADHD. Indeed, irritability [80] and aggressive behaviors [66] can be reduced by learning how to modify hostile interpretation bias and to improve emotion recognition. For instance, the aim of CBT programs for irritability and anger problems is to reduce hostile interpretations [17], to improve emotional regulation skills [29], anger management [19], and the overall functioning of children [43]. A review of meta-analyses indicates that CBT programs are beneficial for children with anger problems [49] and aggression [82]. Moreover, a systematic review showed that psychosocial interventions have beneficial effects on emotional symptoms (irritability, depression), aggressiveness, and overall functioning in children with ADHD and ED [87]. Several studies on emotional regulation in children with ADHD suggest that intervention allows reducing emotional problems and better regulating negative emotions [38, 39, 74]. However, few studies specifically focused on the management of aggressive behavior and anger in children with both ADHD and ED, although these are the most important manifestation of ED. A combination of a self-control program and Anger Control Training reduces aggressive behavior in children with ADHD [64]. Masi et al. [54] reported that in children with ED and disruptive behavior disorders (including 25% with ADHD), a multimodal treatment program led to a modest to significant improvement in the “Aggressive behavior” subscale of the Child Behavior Checklist (CBCL), depending on ED severity.

Battagliese et al. [12] showed that CBT is effective for aggressive behaviors only when interventions are delivered to both parents and child. Similarly, most psychosocial interventions that are effective on ED also included a parent program (Fernández [28, 38, 39, 93]). A meta-analysis indicated that behavioral parent training is effective for reducing oppositional and aggressive behavior in children with ADHD [31]. Behavioral and cognitive behavioral parent programs help parents to manage their child’s behavior by teaching behavioral and cognitive behavioral techniques [98]. According to previous studies [44, 90], parents learn to identify the triggers of anger, to predict problematic behaviors, and to cope with their child’s emotional reactions. Moreover, an emotion socialization program for parents allowed children with ADHD to develop emotional skills and had a protective effect toward comorbidities [39]. As children learn emotional regulation strategies by observing their parents, the parents’ role is crucial to help them to adjust their emotions and acquire emotional skills [16].

Many studies have shown the positive effects of psychosocial interventions on ED, particularly on aggressive behavior and anger, but they have many limitations. Indeed, most studies were uncontrolled, used different measurements, and had small and heterogeneous samples [73]. As there is no validated treatment for ED [41], particularly for children with ADHD and ED, interventions to help children with ADHD and ED to recognize and cope with their emotional problems should be targeted to allow them to reduce the frequency and intensity of their emotional reactivity [71].

This randomized trial compares the effects of a parent/child CBT program and a theater-based intervention (TBI) on aggressive behavior in children with ADHD and ED. This trial proposes an intervention to manage ED in children with ADHD and their parents, and includes an active control group (TBI), differently from most randomized controlled trials [87].

### Objectives {7}

The main objective of this study is to evaluate the effectiveness of a parent/child CBT program compared with a TBI on aggressive behavior in children with ADHD and ED, 6 months after the intervention end.

Secondary objectives are to evaluate the CBT impact on the child’s socio-communicative capacities, quality of life, executive functions, and overall functioning and on parental stress effects) and at month 6 post-intervention.

*The hypotheses of this study are as follows:*

1. The CBT program will lead to a statistically higher reduction of the “Aggressive behavior” sub-score compared with the TBI program at 6 months post-intervention;
2. The CBT program will improve the quality of life in children and their parents, overall functioning, emotion dysregulation, and parental stress and depressive symptomatology at 6 months post-intervention;
3. CBT will improve executive functions measured with the “Behavioral Control Index” of the Behavioral Rating Inventory of Executive Function (BRIEF) questionnaire.

### Trial design {8}

The study described in this protocol is a randomized controlled trial to evaluate the superiority of a CBT program for children with ADHD and ED compared with the TBI program. Sixty-eight children with ADHD and ED will be randomized (1:1) in the CBT or TBI group. Randomization will be stratified by school level and by the psychopathological disorder severity using the Child Behavior Checklist-A-A-A (combination of “Aggression,” “Anxiety/Depression,” and “Attention” subscales). Children will be divided in groups according to their school level: primary school (younger than 11 years) and middle school (11–13 years of age). This classification is required because the situations encountered in everyday life are not the same in middle and primary school.

## Methods: participants, interventions and outcomes

### Study setting {9}

Patients will be recruited at the child psychiatry and neuropediatric units of Montpellier University Hospital. Information leaflets are distributed by mental health professionals to families during consultations in the outpatient services. Full study information is given to parents and children by the investigator before the signature of the informed consent.

### Eligibility criteria {10}

#### Inclusion criteria

Inclusion criteria are as follows: (1) child/adolescent aged between 7 and 13 years; (2) diagnosis of ADHD (based on the DSM-5 diagnostic criteria) assessed with a structured clinical interview (Kiddie-Schedule for Affective Disorders and Schizophrenia Present and Lifetime version; K-SADS-PL), (3) a score ≥ 180 at the CBCL A-A-A when combining the “Aggression,” “Anxiety/Depression,” and “Attention” subscales; (4) child/adolescent followed at Montpellier University Hospital; and (5) families covered by the national healthcare insurance.

#### Exclusion criteria

Exclusion criteria are as follows: (1) child with delayed development or severe language disorder or autistism spectrum disorders; (2) parents who do not speak French; (3) lack of informed consent and assent by the parents and child for their participation in the study; and (4) child not living with at least one parent.

Delayed development was clinically assessed, but in a majority of cases, children also have psychometric tests to help exclude differential diagnosis and respond to administrative requirements for school compensations.

The majority of children included in this study follow regular school. However, children in specific structure school settings for disruptive behavior Educational and Pedagogical Therapeutic Institute will be accepted in this trial if they respond to eligibility criteria.

Study exit criteria are as follows: (1) participants lost to follow-up and (2) consent withdrawal by the parents. Each study exit will be explained and described in detail.

### Who will take the informed consent? {26a}

Informed consent and assent are obtained from the parents and children, respectively, before starting any trial-specific procedure. All participants are advised that participation in research is entirely voluntary and that they can withdraw their participation at any time. Families are not paid for their participation. The child’s psychiatrist or pediatrician does the first presentation of the study. The protocol is then explained in detail by the clinical investigator before the signature of the informed consent by parents and child.

### Additional consent provisions for collection and use of participant data and biological specimens {26b}

In this trial, it is not excluded that collection of data be used for ancillary studies depending ethical authorization.

## Interventions

### Explanation for the choice of comparators {6b}

In this trial, two presumably active groups are compared: the CBT group based on the program “How to improve anger and frustration management” (« Mieux gérer sa colère et ses frustrations », [59]), and the TBI group in which a theater-based group activity is proposed to develop verbal and reciprocal communication, theory of mind, self-awareness of emotions, and interpersonal trust [23, 33, 37]. TBI improves social skills, socio-cognitive functioning, and social interactions in children [24]. TBI also improves self-esteem and shows medium effects sizes for anxiety and depression reduction [45].

TBI was chosen as active comparison group, because the objective of our study is to evaluate the primary and secondary outcomes in two potentially effective groups, while controlling for non-specific effects. In both groups, conditions are similar: duration and number of sessions, group location and setting, recruitment procedures. Differences concern only on the program content (CBT versus TBI).

In this trial, we have chosen to compare two possibly groups, because it was complicated to offer active group versus treatment as usual (TAU) to children with major consequences of their difficulties in daily life. Children in the TAU group would have waited until the end of the study, i.e., approx. 1 year before being able to benefit from a possibly active group.

Both groups can have positive effects on aggressive behaviors in children with ADHD and emotion dysregulation. But, we suppose that the positive effects of CBT group continues after the end of intervention as the emphasis lies on learning new skills, we think this may not be the case in the TBI group, in which the focus is more experiential. In the CBT group, children learn techniques to manage their emotions for which they need time to appropriate and apply them in everyday life, while in the TBI group, it is not the case. That is why, we compare the two active groups between initial visit and at 6 months after the end of intervention and not at the end of intervention.

### Intervention description {11a}

#### Cognitive Behavioral Therapy group

The CBT group is based on the manualized program “How to improve anger and frustration management” (“Mieux gérer sa colère et ses frustrations,” [59]). The CBT program « Mieux gérer sa colère et ses frustrations » is based on the Anger Coping Program [51]. The program has demonstrated effectiveness with a reduction of aggressive behaviors and an improvement of self-esteem [50]. Similar programs based on cognitive behavioral therapy showed that these interventions allow improving aggressive behaviors [82].

This program has been originally designed for impulsive and aggressive children at school [56]. Afterwards, it has been adapted to children with ADHD and has been evaluated several times. A first study carried out on children with Opposant Defiant Disorder (ODD) showed that parents reported a reduction of externalizing behaviors in their child. Teachers highlighted significant improvement of self-control, but no difference on externalizing behaviors. A second study showed satisfaction and feasibility of this program in children with ADHD. Families reported a generalization of techniques in daily life, a reduction of conflict during homework and a better self-control in their child with ADHD [57]. In a study, this CBT program was combined with a parent management training (« Mieux vivre le TDAH à la maison », [60]) and showed an improvement of social skills in children with ADHD, a reduction of parental stress, and a better parent-child relation [58].

Sessions of the CBT program have been developed from social cognitive model of anger and aggression [47] in order to explain that the way by which the child evaluates the situation as well as own emotional and physiological reactions generate aggressive responses. In this model, parents play a crucial role in maintaining and reinforcing of aggressive behaviors in children as well as in characteristics of their child [52].

The program consists of 1-h sessions (one per week; 15 in total) led by a psychologist and a caregiver (nurse or educator) both trained in the management of behavioral disorders and ADHD. They also have in-depth knowledge of CBT that they practice regularly.

During the program, children learn to identify situations that cause anger and frustration, but also techniques to manage negative emotions. Each session is organized as follows: reminder of the previous session by correction of the task done at home, presentation of the theme of the session with practice in the form of role-plays or exercises, presentation of inter-session tasks, and session evaluation. Children evaluate each session on the following criteria: interest and pleasure in the proposed activities, quality of the documents, ease and usefulness of the session in everyday life. Children must achieve a behavioral goal during the session. This challenge is individualized and focuses on each child’s specific difficulties. At the end of the session, the child evaluates whether he has achieved the goal. If the challenge is reached, he/she obtains a reward. Children learn that every effort deserves a reward. Several studies have shown that children with ADHD are particularly sensitive to the reward system and have emotional aversion to delay [10].

During the program, children learn how to identify signals of anger at the body level, triggering situations, behaviors, and thoughts that appear when they are angry. Children also learn ways to diminish angry outbursts, to prevent their increase (e.g., through relaxation, activity practice, thinking about something else), and to create a repertory of solutions to solve problems. They learn to interpret social situations and the importance of thoughts in triggering and maintaining anger. During the final sessions, children learn techniques to peacefully solve conflicts with others and to deal with the anger of others, whether or not this outburst is justified.

#### Theater-based intervention (TBI)

TBI is a role-play activity that is part of our day-care activity schedule. The TBI group participates in 1-h sessions (one per week; 15 in total) led by two mental health workers (nurse or special educator) trained in the management of behavioral disorders, ADHD, and experienced in theater-play. The TBI sessions are not based on a specific program, and their content was developed from existing acting techniques already used in psychiatry (Héril and Mégrier, 2005; Alix and Renard, 2015).

The program objectives are to develop self-control and self-confidence skills, prosocial skills, and appropriate expression of emotions through role-play exercises. During the program, children are also trained in other competences, such as impulsivity control.

Each session includes structured and tailored activities:

- A warm-up phase around active group exercises: children are in motion, while learning how to regulate their impulsivity;
- An individual phase with exercises of improvisation, expression of emotions, and memory;
- A phase of game in pairs that includes several exercise types: role-playing around everyday life, games where children learn how to use prosocial skills and how to work in teams.
- A fun and collective play time that lasts 5 min. This time allows children to let off steam.
- A quiet time: at the session end, children enjoy a time of relaxation or meditation. This moment allows children to refocus on themselves and to learn techniques to manage their emotions. These exercises help to increase physical self-control that can promote better impulse control in children with ADHD [37].

During each session, the program includes sensory games, movement/space games, imitation activities, self-control games, and emotion expression activities.

Each session is adapted according to the individual needs of the children in the group and their age.

#### Parent group

The parent program is the same in both groups because this study objective is to compare the two programs for children and to evaluate their effectiveness on aggressivity in the context of ADHD with ED. Parents’ sessions take place at the same time as the children’s sessions, for convenience.

The parent group is based on a parent training program in which parents are taught behavioral and cognitive behavioral techniques to effectively manage their child’s behavioral problems [98]. There are many manual programs, such as Webster-Stratton’s Incredible Years program [94] and “How to improve living with ADHD at home” (“Mieux vivre le TDAH à la maison”, [60]). Previous studies have shown that parent management training improves childhood behavior problems, allows the development of positive parenting competences, and reduces parental stress [34]. Parents learn techniques to manage their child’s tantrums through videos and information sheets based on the experiences described by other parents. One session was added to this program on psychoeducation about ADHD and ED (session 1). In sessions 2 and 3, parents learn techniques on how to reinforce positive behaviors in their child (e.g., token system, special moment, encouragement…). Often parents of children with ADHD and ED tend to have conflictual and negative relationships with their own child and put in place coercive systems. In sessions 4 and 5, parents receive psychoeducation on anger (e.g., how to identify signs of anger, triggers) and learn several techniques for outburst management (e.g., to ignore minor disruptive behavior, to encourage emotion verbalization by their child). In session 6, parents learn problem-solving techniques, and techniques that allow parents and child to calmly discuss about conflicts and find solutions that are acceptable to both of them. In session 7, parents are taught several techniques to help their child manage their frustration. In session 8, a parent is invited to share a problem encountered in their daily life with their child. The other parents in the group can ask questions to analyze the problem. Then, solutions are proposed, and a solution that seems effective is tried at home. This session allows putting into practice the techniques learnt during the program.

The program is animated by both psychologist and caregiver (nurse or educator). Each session lasts approximately 1 h and it is carried out every second week. Between sessions, parents are asked to implement the acquired techniques in their everyday life. At the next session, sometime is dedicated to discuss their implementation and the difficulties encountered in order to adjust the techniques to the individual needs.

### Criteria for discontinuing or modifying the allocated interventions {11b}

Criteria for discontinuing the allocated interventions are (1) disruptive behavior disorder that does not allow the child to benefit from the intervention; (2) behavior not stabilized by drugs for children under treatment; and (3) aggressive behavior of one participant toward the others.

The protocol does not allow modifying the allocated intervention.

### Strategies to improve adherence to interventions {11c}

To improve adherence to the intervention protocols, behavioral goals are defined to increase motivation. These challenges are individualized and focus on each child’s specific difficulties. At the session end, children must evaluate whether they have achieved their challenge. If the challenge is reached, they obtain a reward. Children learn that every effort deserves a reward.

During the study, participants will be contacted by telephone if absent during two consecutive sessions to know the reasons of their absence. Between sessions, caregivers are available by telephone or email to answer any questions from the families.

### Relevant concomitant care permitted or prohibited during the trial {11d}

During the interventions, pharmacological treatments are permitted. Moreover, pharmacological treatments can be modified if they are no longer appropriate. It is possible to begin a drug treatment during the trial if behavioral symptoms become too severe and have a negative impact on the child’s participation and implication in the group. The maintenance of other usual care components is authorized.

It is recommended that parents do not participate in other parental management training programs, because of similarities between the interventions proposed in the trial and those usually offered by care services.

### Provisions for post-trial care {30}

A participant may exit the trial for the following reasons: severe behavioral problems not sufficiently stabilized to allow the participant to benefit from the program, strong opposition to participate in the group, and inadequate behaviors (physical or verbal aggression against other participants or caregivers) that interfere with the proper functioning of the group. When a child behaves inappropriately (opposition, verbal or physical aggression, provocation…) during a session, a discussion time is offered with the child, parents, and caregivers. The objective is to find a solution together to solve the problem. This exchange may be enough to motivate the participant and stop the inappropriate behavior. Concomitantly, the child’s psychiatrist will be contacted and informed of the situation. He/she might decide to meet the child to modify the pharmacological treatment and to re-motivate him/her. Nevertheless, if the problematic behaviors persist and affect the group, the child exit from the program is decided together with the child, the family, and the psychiatrist.

### Outcomes {12}

Primary and secondary outcomes will be evaluated in the two groups (TBI and CBT) at the inclusion visit (baseline), at the end of intervention, and at month 6 after the end of the intervention.

The primary judgment criterion is the variation of the “Aggressive behaviors” score of the CBCL between baseline and month 6 post-intervention. The initial evaluation will be on outcomes at month 6 post-intervention; because we hypothesize that the effectiveness of such programs is observed several months after their end. Usually, it takes time for children and parents to assimilate and routinely implement the new techniques and also to observe the intervention benefits in the daily life. This will allow also assessing whether the beneficial effects of the interventions are maintained in the long term.

Secondary judgment criteria include also the variation between baseline and month 6 post-intervention of the CBCL A-A-A score, CBCL-Internalizing and CBCL-Externalizing scores, KIDSCREEN-27, Parental Quality of Life and Developmental Disorder (Par-DD-Qol), Children’s Global Assessment Scale (C-GAS), Parenting Stress Index-Short Form, 4^th^ edition (PSI-4-SF), Beck Depression Inventory-II (BDI-II), and Strengths and Difficulties Questionnaire (SDQ) and the SDQ-dysregulation profile (SDQ-DP).

Exploratory analyses will be realized and will concern the variation of primary and secondary judgment criteria between baseline and at the end of the intervention. Exploratory analyses will be realized to identify possible mediators concerning personality traits on therapeutic effects (response to treatment at T2).

Other data will be collected during the initial visit: presence or absence of psychiatric disorders in children using the K-SADS-PL diagnostic tool, sociodemographic data, anamnestic data, and medication history. Pharmacological treatments will be recorded at each visit because the introduction of a new drug or the modification of posology may influence the research outcomes. Indeed, ADHD usual treatment effectively reduces ED ([28, 46, 86]; Gamli et al., 2018 [97];). The Hierarchical Personality Inventory for Children (HiPIC) will be completed at the end of the intervention to limit the duration of the initial visit.

Means, standard deviations or frequencies, median and percentages will be calculated in order to describe sociodemographic and clinical variables of the sample as well as primary and secondary outcomes.

### Participant timeline {13}

Outcome measures are collected at pretreatment (T1), just after treatment end (T2), and at month 6 post-treatment (T3) (Table 1). Data for the primary and secondary outcomes will be collected at child psychiatry unit of Montpellier University Hospital (MPEA Saint Eloi). The child presence is not required for all the measures collected during these assessments.

**Table 1:** Visits and data acquisition during the trial

Timepoint	Study period			
	Enrollment	Before the intervention (T1)	At the intervention end (T2)	Six months after the intervention (T3)
**Enrollment:**				
Eligibility criteria	X			
Child Behavior Checklist (CBCL)	X			
Informed consent	X			
Randomization	X			
**Assessments:**				
Structured clinical interview (K-SADS-PL)		X		
*Parent-rated measures*				
Child Behavior Checklist (CBCL)		X	X	X
Strengths and Difficulties Questionnaire (SDQ-Par)		X	X	X
Behavioral Rating Inventory of Executive Function (BRIEF)		X		X
Hierarchical Personality Inventory for Children (HiPIC)			X	
Kidscreen-27		X	X	X
Parental Quality of Life and Developmental disorder in their children (Par-DD-Qol)		X	X	X
Parenting Stress Index 4^th^ edition (PSI-4-SF)		X	X	X
Beck Depression Inventory-II (BDI-II)		X	X	X
*Child-rated measures*				
Kidscreen-27		X	X	X
*Teacher-rated measures*				
Strengths and Difficulties Questionnaire (SDQ-Teacher)		X	X	X
*Clinician-rated measures*				
*Children’s* Global Assessment Scale (C-GAS)		X	X	X
**Satisfaction questionnaires:**				
*Parent-rated measures*				
Satisfaction questionnaire on the parent program			X	
Satisfaction questionnaire concerning the child program			X	
*Child-rated measures*				
Satisfaction questionnaire on the child program			X	

#### Pretreatment visit (T1)

Participants and their parents will participate in a visit to determine their eligibility (Enrollment, Table 1). Then, the T1 visit takes approximately 2 h, because the diagnostic interview (K-SADS-PL) is carried out systematically with the parents to confirm the diagnosis of ADHD and the presence of comorbidities. During this visit, parents are interviewed by clinicians for clinical ratings of their child’s overall functioning impairment (C-GAS). Children and parents will also complete self-report questionnaires (see Table 1). A questionnaire (SDQ-Teacher) is completed also by the child’s teacher. This questionnaire is either given to parents who will directly transmit it to the teacher, or sent by mail.

#### Visit at treatment end (T2)

After the last session, questionnaires are sent to the parents for completing them at home and bring them back at the T2 visit. Children and parents will complete the same self-report questionnaires as at T1, but for the HiPIC and the satisfaction questionnaires (only at T2), and BRIEF (only at T1) (Table 1). During the T2 visit, parents are interviewed by a clinician to assess the child’s overall functioning (C-GAS). This visit does not exceed 30 min. If needed, questionnaires can also be completed with the help of a researcher. The SDQ-Teacher questionnaire is again completed by the child’s teacher

#### Visit at month 6 post-treatment (T3)

This follow-up visit determines whether the intervention effects persist at month 6 after the intervention end. Children and parents will be interviewed by a clinician to complete the C-GAS. Before the visit, they will complete the same self-report questionnaires as at T1 (at home) (Table 1). This visit takes approximately 30 min.

### Sample size {14}

As ED in children with ADHD has been only recently studied, it is difficult to find literature data for calculating the number of subjects required (particularly for assessing the changes in the “Aggressive behaviors,” “Anxiety/Depression,” and “Attentional Problems” scores of the CBCL scale).

The study by Masi et al [54]. reported a change in the “Aggressive behavior” score of the CBCL from 68.3 to 65.5 in the context of the non-comparative evaluation of an intervention similar to the one assessed in the present trial. The standard deviation of the score at the baseline was 5.4.

By making the reasonable assumption of a slightly more effective intervention than what reported by Masi et al [55]., a CBCL score of 65 in the intervention group and 69 in the control group, with a common standard deviation of 5.5, can be expected at month 6 post-intervention. To highlight such a difference with a power of 80% and an alpha risk of 5%, 31 subjects per group need to be included. By increasing this number by 10% to take into account participants lost to follow-up, the total number of subjects to be included in the study is 68 (34 per group).

### Recruitment {15}

Patients will be recruited at the Child Psychiatry and Neuropediatric units of Montpellier University Hospital. Information leaflets will be distributed by psychiatrists and may be posted electronically to families. Researchers will give presentations on this trial to the general public and mental health professionals.

## Assignment of interventions: allocation

### Sequence generation {16a}

After verification of the eligibility criteria, family’s information, and consent signature, each participant will be randomized. Intervention allocation will be done using a computer-based random number generator. Randomization will be performed as soon as the number of participants is reached to start a session (one CBT group and one TBI group) with a 1:1 ratio.

Randomization will be stratified by school level (primary vs middle school) and severity of psychopathological disorders using the CBCL A-A-A score. Children will be divided in groups according to their school level (< 11 years, and 11–13 years of age).

The investigator in charge of the study assessments is blinded to the group allocation.

### Concealment mechanism {16b}

Randomization is done by the Department of Medical Information of Montpellier University Hospital using the Capture System software (Clinsight).

### Implementation {16c}

If participants are interested in the trial, psychiatrists or pediatricians give information and enroll them. Then, participants are contacted by telephone by the investigator. The psychologist informs about the study, verifies that families are aware of the implications of participation in a trial, and proposes an appointment for the initial evaluation. She verifies that children are motivated and willing to participate in the trial. Indeed, often, parents register their child without asking their opinion, while it is important that they are involved and motivated in order to benefit from the intervention. After the initial evaluation, participants are assigned to an intervention group by the research coordinator after randomization.

## Assignment of interventions: blinding

### Who will be blinded {17a}

The research psychologist who performs the baseline and outcome assessments will be blind to the treatment conditions. Due to the nature of the interventions, it will not be possible for the researchers who administer the intervention to remain blind. Researchers who administer the intervention will not be involved in the collection of the outcome data to avoid bias in measuring results.

Given the study design, parents and children are not blind and are informed about the group allocation after randomization.

### Procedure for unblinding if needed {17b}

There is no procedure for revealing a participant’s allocated intervention during the trial, because it is not a protocol targeting a pharmacological treatment. Therefore, it did not seem necessary to provide circumstances for and an unblinding protocol.

## Data collection and management

### Plans for assessment and collection of outcome data {18a}

Outcome data will be collected by a research psychologist with a case report form (CRF). Data will be verified by supervised psychology students to detect errors in the collected data. Finally, data will be verified by a methodologist who will process all statistical data.

The case report form includes the following:

- Initial inclusion: inclusion and non-inclusion criterion; date of signed consent, inclusion in the study and randomization; data of all questionnaires, sociodemographic characteristics (marital status, socioeconomic status, education attainment), and clinical characteristics of children (age, sex, school level, comorbid disorders, drug treatments, rehabilitation therapy) and parents (somatic and psychiatric pathologies);
- Participations in intervention: at each session, we will note the date, the presence or absence of parents and child, the reason for absence, and the name of present parent (mother, father or other);
- Visit at the end of intervention (T1): data of primary and secondary outcomes; life events, introduction or modification in drugs treatment; side effects;
- Visit at 6 months after intervention (T2): data of primary and secondary outcomes; life events, introduction or modification in drugs treatment; side effects.

It is possible to provide French version of case report form on a request of the Editorial Office.

#### Primary outcome

##### Child Behavior Checklist (CBCL [3];)

The CBCL is a 118-item parent-completed questionnaire to measure emotional and behavior problems in 4- to 18-year-old children in the past 6 months. Parents rate each item as 0 (not true), 1 (somewhat or sometimes true), or 2 (very true or often true). The CBCL includes eight syndrome scores: Anxious/Depressed, Withdraw/Depressed, Somatic Problems, Social Problems, Thought Problems, Attention Problems, Rule Breaking, and Aggressive Behavior. A computer program calculates the *T*-scores for each scale. Raw scores are converted to gender- and age-standardized scores. A *T*-score of 50 indicates average functioning and standard deviation is every 10 points.

The objective of the primary outcome is to assess the effectiveness of the CBT program on the “Aggressive behavior” score (i.e., primary outcome) of the CBCL A-A-A. The CBT program should allow reducing aggression.

The “Aggressive behavior” sub-score contributes to the CBCL-DP and “Deficient Emotional Self-Regulation” (CBCL-DESR). The two profiles are the sum of the CBCL “Anxious/Depressed,” “Attention Problems,” and “Aggressive Behavior” (A-A-A) syndrome *T*-scores [3]. The CBCL-DESR is present when the sum of the CBCL A-A-A scores is ≥ 180, but below 210 (*T*-scores > 60). The “Dysregulation Profile” is present when the sum of the CBCL A-A-A scores is ≥ 210 (2 SD; *T*-scores > 70). These profiles represent a continuum of the emotional and behavioral dysregulation severity [14, 55]. They allow defining an ED phenotype and are a risk marker of a persisting deficit of self-regulation of emotion and behavior [40]. CBCL-DP and CBCL-DESR allow identifying a group of children with disruptive behavior [4] and at risk of severe dysfunction [30, 40, 79]. The CBCL-DP and CBCL-DESR profiles are associated with higher risk of comorbidities [27, 40, 55], hospitalization in psychiatry services [27], and development of inappropriate personality traits [67] in adulthood. The CBCL-DESR profile is a predictor of increased risk of opposition defiant disorder, conduct disorder, and anxiety disorders [79]. The CBCL-DP predicts high risk of mood disorders during adolescence [54].

#### Secondary outcomes

The trial includes several secondary outcome measures, and also the clinician ratings of impairment (C-GAS). Moreover, parents complete questionnaires about their child to evaluate:

- Global functioning (SDQ, C-GAS)
- Executive functions (BRIEF)
- Personality traits (HiPIC)
- Quality of life (Kidscreen-27).

Parents also complete questionnaires to evaluate:

- Parental stress (PSI-4-SF)
- Parental depression (BDI-II)
- The impact of their child disorders on the quality of family life (Par-DD-Qol).

Secondary outcomes include also teacher-report measures of the child’s global functioning (SDQ-Teacher), and child-report measure of the quality of life (Kidscreen-27).

The study objectives are to evaluate the CBT program impact on the socio-communicative capacities, emotional and behavioral self-regulation capacities, quality of life of child and family, overall functioning of child, and parental stress/depression in the short term (intervention end) and also at month 6 post-intervention. Finally, the personality profiles of children with ADHD will be analyzed to determine whether there are common personality traits in children with ADHD and ED.

##### French version of the Strengths and Difficulties Questionnaire (SDQ-Fr, Goodman, 1997)

SDQ-Fr is a standardized questionnaire completed by parents or teachers that allows a brief emotional and behavioral screening of 2- to 17-year-old children and adolescents (http://www.sdqinfo.com). The SDQ-Fr includes 25 items divided in 5 subscales with 5 items/each to assess emotional symptoms, conduct problems, hyperactivity/inattention, peer relationship problems, and prosocial behavior. The questionnaire is completed on paper in 5–10 min.

The SDQ-Fr allows obtaining a “dysregulation profile” (SDQ-DP). It is a combination of two items from the Emotional symptom subscale (“Many worries, often seems worried,” “Often unhappy, down-hearted or tearful”), two from the Conduct problem subscale (“Picked on or bullied by other children,” “Steals from home, school or elsewhere”), and one from the Hyperactivity/Inattention subscale (“Restless, overactive, cannot stay still for long”). A study suggested that children with ADHD and SDQ-DP display more angry reactions and are less capable to control their anger [22]. The SDQ-DP allows identifying children with ADHD at risk of severe difficulties in overall functioning. A study showed that SDQ-DP is a predictor of developing psychiatric disorders in the next 4 years [91].

##### Kidscreen-27 (KIDSCREEN Group, 2006)

Kidscreen-27 is a questionnaire that evaluates the generic health-related quality of life (HRQol) in 6- to 18-year-old children. The Kidscreen-27 items are derived from the Kidscreen-52 questionnaire. There are two versions, one completed by parents (or primary caregivers) and the other by children. Children evaluate the subjective perception of their well-being during the last week. Kidscreen-27 comprises five domains: “Physical well-being,” “Psychological well-being,” “Autonomy and parents,” “Social support and peers,” and “School environment”. The responses are scored on 5-point Likert scales for frequency (never to always) or intensity (not at all to extremely). Kidscreen-27 has been translated into 36 languages. Kidscreen-27 scores are converted to *T*-values (standardized mean = 50, standard deviation = 10). Higher scores indicate good HRQol and well-being [70].

##### Parental Quality of Life and Developmental Disorder in their children (Par-DD-Qol [13];)

The Par-DD-Qol is a parent-completed 17-item questionnaire to assess the impact of their child’s chronic disabilities on the parental quality of life (Qol). It is particularly adapted to parents of children with neurodevelopmental disorders, because these are chronic conditions that impact the parents’ Qol. The questionnaire includes several dimensions: “Emotional,” “Daily Disturbance,” and “Global Qol.” It is adapted from the Par-ENT-Qol [13] used in the general population with chronic ear, nose, and throat (ENT) infections. Responses are scored on a 5-point Likert scale (not at all to very much). Several studies ([8]; Raysse, 2011) showed a reliability coefficient higher than 0.82 (Cronbach’s alpha) for each dimension. A score below 40 indicates “no impact” on the parents’ Qol, a score between 40 and 57 a “moderate impact,” and a score higher than 57 a “high impact.”

##### Behavioral Rating Inventory of Executive Function (BRIEF; Gioa, Guy & Kentworthy, 2000)

BRIEF is a parent- or a teacher-completed 86-item inventory to assess the executive functioning of 5- to 18-year-old children at home and in school environments using a three-point scale for frequency (never to always). The instrument includes eight scales that measure executive functioning: initiate, work memory, plan/organize, organization of materials, and monitor (forming the metacognition index [MI]), and inhibit, shift, and emotional control (forming the behavioral regulation index [BRI]). The BRI assesses the ability to use appropriate inhibitory control to shift cognitive sets and modulate emotions and behaviors. The MI assesses the ability to use working memory to initiate, plan, and sustain future-oriented problem solving [61]. BRIEF includes two validity scales (Negativity and Inconsistency of responses) to identify the parents’ response styles and to validate the questionnaire quality. A *T*-score of 65 indicates clinically significant executive function impairment. Gioia et al. (2000) reported that the BRIEF reliability coefficient ranges from 0.80 to 0.97 for the two forms (Parent and Teacher), except for two scales (“Initiate” and “Shift”).

##### Parenting Stress Index, 4th edition, short form (PSI-4-SF, [1])

The PSI-4-SF is a 36-item parent-completed questionnaire to measure the parental stress and to detect difficulties in the parent-child dyad. This is defined as a state of psychological malaise in parents related to the parent-child relationship. Responses are provided on a 5-point Likert scale (totally disagree to totally disagree). The questionnaire includes three subscales (“Parental distress,” “Dysfunction in parent-child interactions,” and “Difficulties in children”) to assess the factors that may influence the level of stress experienced by parents in their relationship with their child. The first subscale “Parental distress” allows measuring the distress experienced by parents in the exercise of their role. The second subscale “Dysfunction in parent-child interactions” measures the parents’ satisfaction of their relationship with their child and whether the child meets their expectations. The third subscale “Difficulties in children” assesses the parents’ degree of distress due to their child’s difficult behavior. A score between the 85th and 89th percentile indicates high stress level, and a score ≥ 90th percentile is clinically significant. Abidin [2] reported a reliability coefficient of 0.84 (test-retest, 6 months) and internal consistency coefficient of 0.95 (Cronbach’s alpha) for the total score. The three subscales have internal consistency coefficients of 0.90, 0.89, and 0.88, respectively. PSI-4-SF is strongly correlated with the original version of the PSI-4, with a correlation coefficient of 0.98 for the Total Stress Scale.

##### Beck Depression Inventory-II (BDI-II; Beck, Steer & Brown, 1996)

The BDI-II is the most popular screening instrument for depression in adolescents and adults. It is a 21-item self-report questionnaire that examines the behavioral, emotional, somatic, and cognitive symptoms of depression in the past 2 weeks. Each item is rated on a 4-point Likert scale (ranging from 0 to 3), reflecting the symptom severity. Scores from 0 to 13 indicate “Minimal depression,” scores from 14 to 19 “Mild depression,” scores from 20 to 28 “Moderate depression,” and scores higher than 29 indicate “Severe depression.”

Wang and Gorenstein [92] reported a mean alpha coefficient of 0.9 (ranging from 0.83 to 0.96) and excellent coefficients of retest reliability (0.73 to 0.96). The BDI-II is valid in different cultures and presents strong psychometric properties (Beck, Steer & Brown, 1996).

##### Hierarchical Personality Inventory for Children (HiPIC, Mervielde & De Fruyt, 1999)

HiPIC is a 144-item parent-completed questionnaire to obtain a profile of the personality of 6- to 12-year-old children according to a five-factor model. Responses are scored on a 5-point Likert scale (from “Very untypical” to “Very typical”). Five personality dimensions are assessed: “Emotional Stability,” “Extraversion,” “Benevolence,” “Conscientiousness,” and “Imagination.” HiPIC is scored on 18 facets composed of 8 items/each that are summed and averaged to obtain the dimensions. The “Extraversion” dimension includes positive emotionality, energy in children, and ease in social situations. The “Emotional stability” dimension assesses negative emotions and reactions toward their environment. The “Conscientiousness” dimension measures the determination and drive to achieve a goal. The “Benevolence” dimension evaluates agreeableness, attitude in relationships, and ability to empathize. The “Imagination” dimension represents creativity, curiosity, and openness to new experiences. Mervielde and De Fruyt (1999) reported a reliability coefficient of 0.70 (Cronbach’s alphas) for each domain and facet.

The objective of the inclusion of HiPIC is to better understand personality profile in children with ADHD and emotion dysregulation. Currently, there is a lack of studies in personality traits in children with ADHD and emotion dysregulation.

In this trial, we did not expect to assess variation of personality traits (HiPIC), but just better understand the overall functioning of children with ADHD and emotion dysregulation and to assess whether personality profiles would predict treatment response. As the HiPIC is a relatively long questionnaire and as personality is considered stable in the study’s time frame, we included it at T1 rather than at baseline.

##### Children’s Global Assessment Scale (C-GAS, [75])

C-GAS is a numeric scale to assess the general functioning in 4–16-year-old children. This scale is used and completed by mental health clinicians. A clinician interviews the child, parents, and school staff to assess the child’s global functioning. C-GAS is adapted from the Global Assessment Scale for adults. Scores are divided in ten categories that range from “Extremely impaired” (1–10) to “Doing very well” (91–100).

##### Schedule for Affective Disorders and Schizophrenia for School-aged Children, Present and Lifetime version (K-SADS-PL [42];; French version Mouren-Siméoni & al., 2002)

K-SADS-PL is a semi-structured diagnostic interview to assess all current and lifetime DSM-IV Axis I mental disorders in 6–18-year-old children. K-SADS-PL is administrated by trained mental health clinicians (master level).

#### Other measures

Demographic information was obtained thanks parents during inclusion visit (T0). For all children, clinical investigator collects gender, age, and school level, and for parents, marital status, academic level, socioeconomic status, and psychiatric/somatic pathologies. Pharmacological treatments were recorded at each visit, because introduction or interruption of medication or modification of posology may influence research outcomes. Indeed, ADHD usual treatment effectively reduces emotional dysregulation [46, 84, 86, 97]. Likewise, life events (e.g. move, death or illness of a family member…) were also listed at each visit, as they could have an impact on results.

### Plans to promote participant retention and complete follow-up {18b}

Evaluation visits will be scheduled at the intervention end (T2) and at month 6 post-intervention (T3) (see Table 1). Two weeks before the intervention end, the psychologist will propose to parents an appointment for the T2 visit. For the appointment of the follow-up visit (T3), families will be contacted by telephone by the psychologist. For families difficult to reach, several telephone calls may be made. The child’s referring psychiatrist will be also contacted to improve data collection (e.g., he/she might call the family to emphasize the importance of completing the questionnaires, refer family to the psychologist after consultation…).

For families unable to attend the visit (health problems…), it might be exceptionally decided to complete the questionnaires and the case report form (CRF) during a telephone conversation. This will be indicated in the CRF.

If parents decide to interrupt their trial participation, the investigator will contact them to know whether they agree to complete the final questionnaires.

To promote participant retention during the program, families will be contacted by telephone in case of absence during two sessions.

### Data management {19}

For each participant, data will be reported in a CRF, first in paper format and then electronically. The used software, Capture System, complies with the FDA recommendations on computerized systems for managing clinical trials.

The data manager will perform additional computerized consistency tests to detect the presence of non-standard, missing, aberrant, or incoherent data. These tests will be executed regularly during the participants’ recruitment and monitoring. Each identified incoherence will be the subject of a request for clarification to the researcher.

Data will be saved by the Clinical Research and Epidemiology Unit (CREU) of Montpellier Hospital and will be stored in ASCII type format.

### Confidentiality {27}

Data will be collected in the CRF only by the research psychologist. Each CRF will be anonymized to respect the participants’ confidentiality.

### Plans for collection, laboratory evaluation, and storage of biological specimens for genetic or molecular analysis in this trial/future use {33}

This trial will not involve collection and storage of biological specimens for genetic or molecular analysis.

## Statistical methods

### Statistical methods for the primary and secondary outcomes {20a}

An intention-to-treat approach will be used to analyze the primary and secondary data from the trial. Participants will be analyzed in their randomization arm and cannot change group during the study.

The primary outcome measure is the changes of the “Aggressive behavior” sub-score between T1 (baseline) and T3 (month 6 post-intervention). A *Z* test will be used to compare the changes of the primary (between T1 and T3) and secondary outcomes (between T1 and T2/T3) in the CBT group and TBI group. In the case of group non-comparability, a multivariate model by linear regression could be carried out to take into account the potential confounding bias.

### Interim analyses {21b}

In this trial, statistical analyses of data will be realized when the statistician will have all data (i.e., when the number of subjects required will be reached and when all visits will have been carried out).

The final decision to finish the trial will be taken by mutual agreement of the principal investigator, the data manager, the research coordinator, and the independent safety monitor. Data will be locked when all data have been checked and all corrections done.

### Methods for additional analyses (e.g., subgroup analyses) {20b}

In the case of non-comparability for one or more parameters, an adjustment will be made on this/these parameter(s) for analysis of the judgment criteria. A multivariate linear regression model might be performed to account for potential confounders.

The protocol does not plan analyses for additional subgroups.

### Methods in analysis to handle protocol non-adherence and any statistical methods to handle missing data {20c}

Analysis of the primary and secondary outcomes will be carried out per protocol for all families who participated in two thirds of the intervention.

For missing data, under the hypothesis of a Missing at Random (MAR) or Missing Completely at Random (MCAR) mechanism, an imputation method using the Markov chain Monte-Carlo method will be used with 15 imputation cycles. The analysis will be performed independently for each complete database and the results will be taken into account for estimating the final parameters and their standard deviations.

### Plans to give access to the full protocol, participant-level data, and statistical code {31c}

On request, participants will be informed about the trial overall results by the principal investigator. Families will not have access to personal data.

## Oversight and monitoring

### Composition of the coordinating center and trial steering committee {5d}

Data monitoring committee is composed of a research assistant who ensures compliance with progress of research protocol and regulatory aspects. Data collection and management and statistical analyses are carried out by Department of Medical Information (DMI) of Montpellier University which is made up of a methodologist, a statistician, and a data manager. Daily, groups are led by psychologists, nurses, and educators. Blind evaluations are carried out by a psychologist who recruit participants, collects data, and leads interventions. Two doctors, including principal investigator, sign consents.

### Composition of the data monitoring committee, its role, and reporting structure {21a}

Data monitoring will be carried out by an independent safety monitor who will ensure the compliance with the trial regulatory aspects and will verify data collection. The safety monitor acts as a representative of the study promotor.

### Adverse event reporting and harms {22}

Due to the intervention types, serious adverse events are not expected. Serious adverse events will be reported by the promotor only if they are directly related to the protocol. Each adverse event will be recorded in the CRF by the principal investigator and will be monitored until resolution or stabilization. The principal investigator will evaluate each event and its gravity and will contact the trial promotor.

### Frequency and plans for auditing trial conduct {23}

Investigators accept to comply with the regulatory requirements of the promoter and the competent authority for a research audit. Audit may be carried out at any stage of the trial, from the protocol development to the publication of results and archival of the data used for the study.

### Plans for communicating important protocol amendments to relevant parties (e.g., trial participants, Page 27 of 40 ethical committees) {25}

All protocol modifications must be submitted to and validated by the Local Human Subject Protection Committee before their implementation. When the protocol modification is accepted by the committee, the research assistant ensures that the changes are implemented accordingly.

### Dissemination plans {31a}

All communication of results must receive the prior agreement of the principal investigator and promoter. Montpellier Hospital is the data owner and must be mentioned as the trial promoter. Regardless of magnitude or direction of effect, data will be published in a scientific review. It is expected that the article with primary outcomes results will be published once all data are validated by the Department of Medical Information composed of data manager, statistician, and methodologist. On request, participants will be informed about the trial overall results by the principal investigator.

## Discussion

This randomized controlled trial wants to investigate whether a CBT program can improve aggressivity in children with ADHD and ED. To this aim, the trial compares the effects of two interventions (CBT and TBI) on emotional and behavioral components in children with ADHD and ED. A parent management program is implemented in both groups. Secondary objectives are to examine the intervention impact on the child’s socio-communicative capacities, quality of life, executive function, and overall functioning at the end of the intervention and after 6 months. The last objective is to examine the intervention effects on parental stress, quality of life, and depressive symptoms. This is one of the first studies that evaluate the short- and long-term efficacy of a psychosocial intervention on aggressive behavior in children with ADHD and ED. Most studies tend to assess effectiveness in the short term (i.e., at the end of the intervention or after 3 months). To our knowledge, no study on psychosocial interventions in children with ADHD and ED assessed the effects of such programs after 6 months.

The results from this trial will be useful for the management of children with ADHD and ED, because this type of interventions should allow children to develop emotional and behavioral self-regulation skills by learning techniques to manage anger (relaxation, resolution problems…). Indirectly, it is expected that the program will promote the development of prosocial skills, leading to a more harmonious relationships with peers and family. For parents, the trial objective is to improve their educative strategies, and also the quality of family life and relations.

The primary limitation of the trial is the lack of tools to detect the intervention effects on ED in children with ADHD. Indeed, most of the available tools for ED evaluation (Bunford, Evans, & Wymbs, 2015), such as physiological, observational, and neuropsychological measures, do not take into account the specificities of subjects with ADHD [32]. Therefore, the subscale “Aggressive behavior” of the CBCL-DP was selected as the primary outcome, because it allows evaluating the degree of severity of aggression in children and adolescents [5] and is a good ED marker.

ADHD represents a public health problem, because it has a significant impact on the children and their family’s daily life and represents an economic burden [48]. Developing effective early psychosocial treatment options for children with ADHD and ED is particularly important in order to prevent developmental trajectories with poor prognosis (social difficulties, comorbidities, persistence of ADHD symptoms), and possibly to limit the need of pharmacological treatments.

## Trial status

Protocol version: August 8, 2018 Version n°3.

Date of recruitment: June, 2017

End of recruitment: September, 2020

The article was submitted after the end of recruitment and not at the beginning: with the co-authors, we favored the implementation of research (doctors' information for recruitment, animation of groups) as well as the writing of a background article that has been published. In addition, due to the pandemic, several participants of the last group dropped out of the study. We had the agreements of the methodologist and the research assistant to extend recruitments and continue the study with another session. Research is currently underway as patients are still participating in groups.

## Abbreviations

ADHD: Attention deficit hyperactivity disorder
CBT: Cognitive behavioral therapy
CRF: Case report from
ED: Emotional dysregulation
TBI: Theater-based intervention
